# Development of a Mobile Application for the Self‐Management of Spinal Cord Injury Patients: Identification and Analysis of Key Requirements

**DOI:** 10.1002/hsr2.71340

**Published:** 2025-11-09

**Authors:** Amir Hossein Daeechini, Azamossadat Hosseini, Reza Rabiei, Saeed Oraee‐Yazdani, Somayeh Paydar, Fatemeh Rahimi

**Affiliations:** ^1^ Department of Health Information Management and Medical Informatics, School of Allied Medical Sciences Tehran University of Medical Sciences Tehran Iran; ^2^ Department of Health Information Technology and Management, School of Allied Medical Sciences Shahid Beheshti University of Medical Sciences Tehran Iran; ^3^ Shohada Tajrish Neurosurgical Center of Excellence, Functional Neurosurgery Research Center, Shohada Tajrish Hospital Shahid Beheshti University of Medical Sciences Tehran Iran; ^4^ Department of Health Information Technology, School of Allied Medical Sciences Kermanshah University of Medical Sciences Kermanshah Iran

**Keywords:** mobile applications, mobile health, self‐management, spinal cord injury

## Abstract

**Background and Aims:**

Spinal cord injury causes numerous complications for those affected by this disability. Using mobile health tools to acquire self‐management skills can help reduce these complications and improve the quality of life of people with spinal cord injury. Therefore, this study aimed to identify and analyze the key requirements, including data and functional requirements in developing a mobile application for self‐management of spinal cord injury patients based on the perspectives of both patients and related specialists.

**Methods:**

A content analysis of previous studies and self‐management guidelines for patients with spinal cord injury was conducted until January 2024. A researcher‐made questionnaire was provided to 20 spinal cord injury patients and 20 specialists to extract key requirements, including data and functional requirements, to develop a mobile application for self‐management of spinal cord injury. The collected data were analyzed using SPSS 26 software.

**Results:**

The thematic categories of “educational information”, “disease and complication management”, and “patient profile” were identified as data requirements, whereas “technical capabilities” were categorized under functional requirements for a mobile application for self‐management of spinal cord injury patients. The key requirements identified in the category of educational information had the highest average score, with a score of 4.63 out of 5, and the patient profile had the lowest average score, with a score of 4.46 out of 5.

**Conclusions:**

The identified key requirements can be used as the first step in determining the educational content and design of a spinal cord injury self‐management application.

**Abbreviations**
mHealth: mobile health
SCI: spinal cord injury

## 1 Introduction

Spinal cord injury (SCI) is one of the most common causes of disability and mortality worldwide, affecting between 250,000 and 500,000 people annually, and is often accompanied by complex disorders. These disorders can lead to severe complications and permanent disability of the person, so these people are 2–5 times more prone to premature death [1, 2, 3].

Traffic accidents and sports injuries are the most common causes of SCI in adults. In addition, falls in the elderly have been identified as the most common cause of SCI. Spinal cord injury can cause partial or complete loss of sensory function or motor control of various organs, including hands and feet [4, 5, 6].

Autonomic dysreflexia, urinary dysfunction and infections, sexual problems, pressure ulcers, depression, and gastrointestinal disorders are among the most common secondary complications for SCI patients. Improper management of these secondary complications can lead to high costs, physical and psychological consequences, and changes in the lifestyle of these people. In other words, 36% of patients with spinal cord injury are re‐hospitalized at least once due to secondary complications related to spinal cord injury [7, 8, 9, 10].

A significant solution to reduce the incidence of secondary complications related to spinal cord injury is the use of self‐management strategies. Self‐management refers to the patient's ability to manage symptoms, treatments, physical and psychological consequences, social aspects of the disease, and lifestyle changes. Self‐management strategies emphasize the key role of people in managing their condition, and the goal of these strategies is to increase people's problem‐solving and decision‐making skills to better manage the disease [11, 12]. A 2014 online survey in Canada revealed that approximately 75% of people with spinal cord injuries considered it very necessary to develop self‐management strategies for people with spinal cord injuries [13].

Currently, advancing self‐management strategies require mobile technologies, which facilitate communication and play a significant role in the rapid development and expansion of innovative technologies in the health industry, leading to a new field of mobile health (mHealth) [14]. The primary goal of using mHealth applications is to help patients manage their health conditions, reduce healthcare costs, facilitate primary care delivery, and provide healthcare services when in‐person visits are impossible [15, 16, 17].

Numerous m‐health apps have been developed to facilitate self‐management skills for individuals with various diseases. These mobile apps are recognized as innovative solutions that provide optimal support to individuals with disabilities, including patients with spinal cord injury. Using mHealth apps enables patients to take control of managing their health and achieve a certain level of empowerment [8, 16].

M‐health apps have been widely embraced by individuals with SCI [9]. Due to the need to pay attention to the needs of these patients, particularly the need for proper education, awareness, and supportive programs for managing multiple complications arising from spinal cord injury these patients are ideal candidates for the development of mobile applications to support their self‐management.

In a similar study [18], which aimed to identify the requirements needed in the design of the self‐care system for the prevention of COVID‐19, demographic data requirements, clinical requirements, self‐management strategies, and technical capabilities were identified and determined as required requirements.

Additionally, in another study by Yazdanian et al., demographic data, clinical data, educational information, disease management, and the app's capabilities and functions were recognized as essential requirements for developing a self‐management app for patients with gastric cancer [19].

A significant gap exists in the availability of comprehensive SCI self‐management applications. Although existing mobile health (mHealth) applications for individuals with SCI have gained some acceptance, most are narrowly focused on specific and limited aspects of SCI management, such as pressure ulcer prevention [20] home exercise [21], or rehabilitation [8]. This study is crucial because there is a need for mobile applications where educational content and technical functionalities are developed based on a thorough needs assessment involving both relevant healthcare professionals and SCI patients themselves. This study aimed to address this gap by identifying and analyzing key data and functional requirements for the development of an SCI self‐management mobile application, incorporating perspectives from both patients and relevant professionals.

## 2 Methods

### 2.1 Study Design

**Figure 1:**
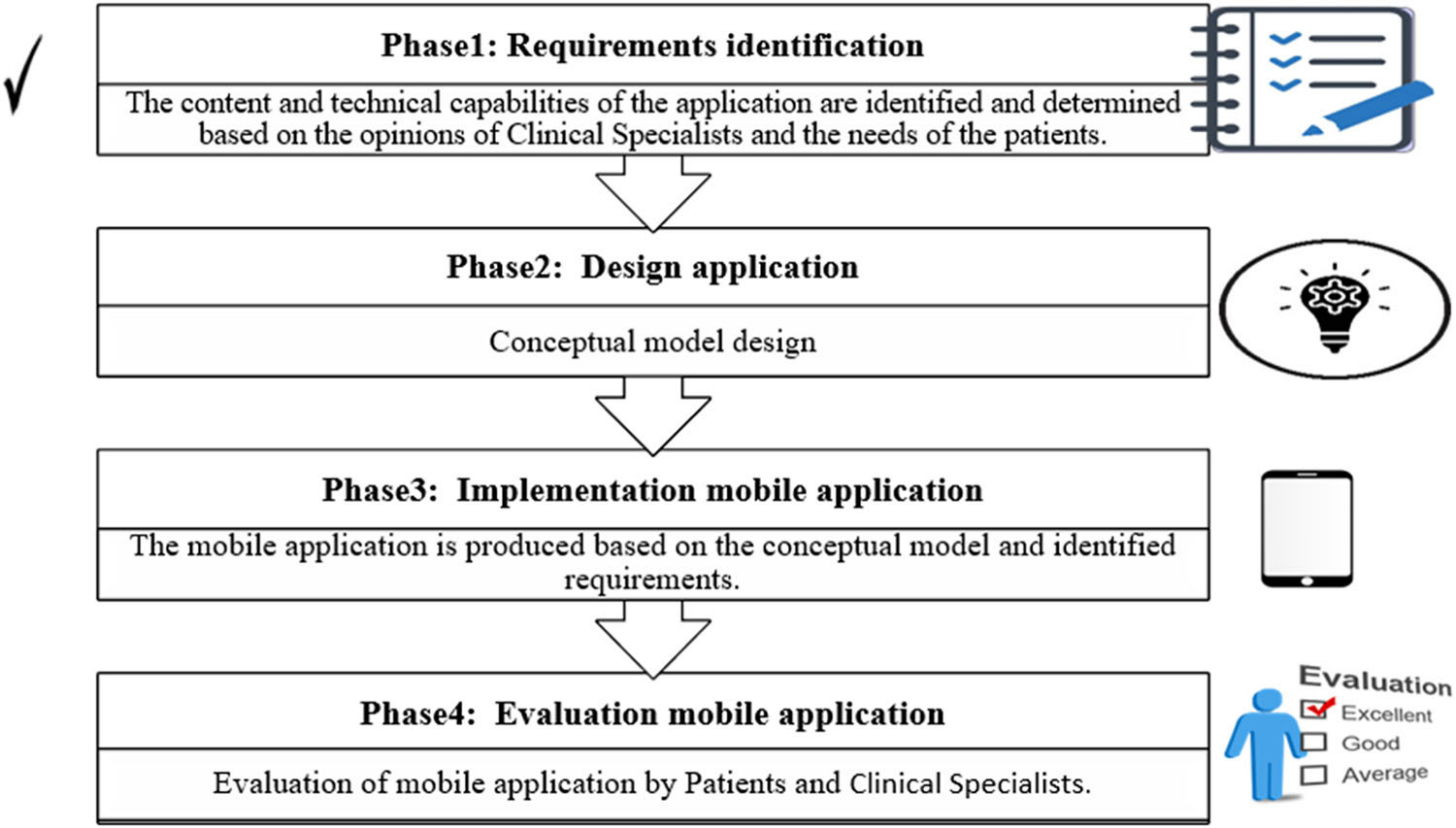
Mobile application development phases.

This applied study was conducted in 2024 at Nasim Clinic in Tehran and Shohada Tajrish Hospital, one of the largest and most equipped educational and treatment centers of Shahid Beheshti University of Medical Sciences in Tehran. The hospital receives numerous patients from diverse cultural and geographical backgrounds daily. The present study covers the first phase of a four‐phase plan to develop a mobile application for self‐management of patients with spinal cord injury (Figure 1).

Figure 1 provides a visual overview of these four phases: (1) requirements identification (the current study), (2) design, (3) implementation, and (4) usability evaluation. Each phase builds upon the previous one, ensuring that the final mobile application is evidence‐based and user‐centered.

**Figure 2:**
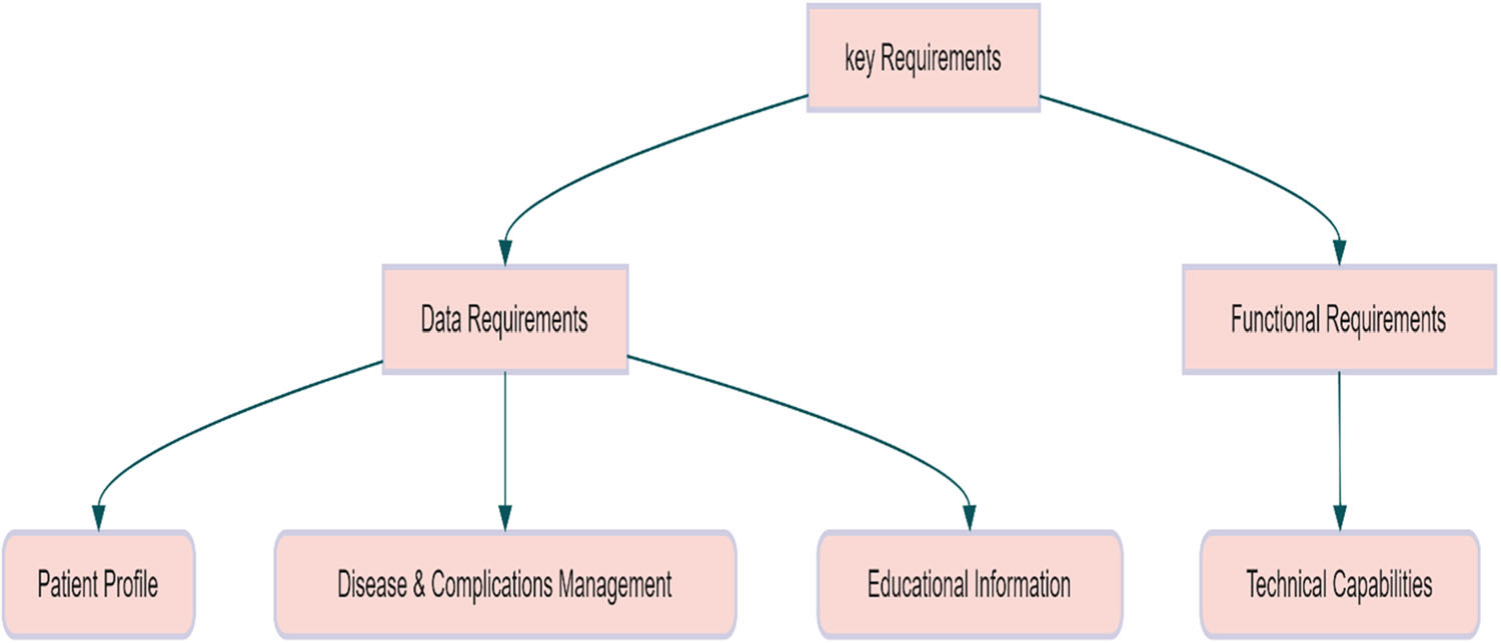
Key requirements are determined in mobile application development.

The specific objectives of this study include identifying and analyzing data requirements in three thematic categories: “educational information”, “disease and complication management”, and “patient profile”, as well as identifying and analyzing functional requirements in the “technical capabilities” category (Figure 2).

### 2.2 Literature Review

A comprehensive literature review was conducted using scientific books, related articles, and specialized guidelines for SCI self‐management to identify the data and functional requirements needed to develop mobile applications for the self‐management of SCI patients. Moreover, databases such as Scopus, PubMed, and Web of Science were searched using the keywords “self‐management”, “spinal cord injury”, “mHealth”, “data requirements”, and “ functional requirements”, along with synonyms for these terms. Studies were screened and selected based on predetermined inclusion and exclusion criteria. Inclusion criteria included: English‐language articles with full text available and sufficient detail on the data and functional requirements of people with spinal cord injury. Conference papers, letters to the editor, review articles, and articles that did not provide sufficient detail on the data and functional requirements of people with spinal cord injury were considered exclusion criteria. After the retrieved studies, the necessary data requirements (content capabilities) and functional requirements (technical capabilities) for developing the mobile self‐management application for SCI patients were extracted into an Excel file. The data requirements were then categorized into three thematic categories: “patient profile”, “disease and complication management”, and “educational information”. The functional requirements were categorized under “technical capabilities”, which refers to the essential technological and functional features required for the mobile application to effectively support self‐management in SCI patients. These categories were derived from recurring themes and requirements highlighted across multiple sources. Table 1 demonstrates the explicit mapping between the findings of the literature review, the identified thematic categories, and the specific items included in the questionnaire. This approach ensured that each questionnaire item was grounded in evidence and addressed the key requirements highlighted in the literature.

**Table 1:** Mapping of literature review findings to thematic categories and questionnaire items.

No.	Themes	Specific areas	Rationale for inclusion	Key literature source
1	Patient profile	Demographic data clinical data	Items were based on demographic and clinical data needs from user‐centered mHealth studies.	[16, 18, 19, 22, 23, 24]
2	Disease and complications management	General information on disease and complications	The items in this section include providing general information about spinal cord injury and the management of its complications.	[8, 9, 16, 23, 25, 26]
3	Educational information	Pressure ulcer Autonomic dysreflexia ADL Nutrition Mental health Bowel and bladder Training FAQ	The items in this section are based on the educational needs of users in the self‐management of spinal cord injury.	[8, 16, 20, 21, 23, 25, 27]
4	Technical capabilities	Reminders Notices App capabilities	The items in this section include the essential technological and functional features required for a mobile application to effectively support self‐management in spinal cord patients.	[16, 20, 22, 23, 25, 28, 29]

### 2.3 Questionnaire Development

The development of the questionnaire was grounded in the thematic analysis of the literature.

Themes formed the framework for the questionnaire design. For each identified theme, relevant items were formulated to capture the specific data and functional requirements mentioned in the sources reviewed. Based on these categories, a researcher‐designed questionnaire (using a 5‐point Likert scale) was developed in Supporting Information S1: Supplementary 1,2. The questionnaire was designed in Persian and consists of 4 sections and 43 items, including “Patient Profile” (10 questions), “Disease and Complications Management” (7 questions), “Educational Information” (17 questions), and “Technical Capabilities” (9 questions). To ensure the content validity of the questionnaire, a specialized panel was assembled, consisting of experts from three relevant disciplines: neurosurgery, health information management, and medical informatics. Panel members were selected based on their academic rank (all were faculty members), professional experience (a minimum of 10 years in their respective fields), and prior involvement in clinical care, research, or technology development related to spinal cord injury or digital health. Specifically, the panel included 10 neurosurgeons, 10 health information management specialists, and 10 medical informatics specialists. After initial review, a subgroup of five experts from each discipline (15 in total) with the most extensive experience in SCI care and digital health participated in the final content validation. Their feedback was used to revise and refine the questionnaire to ensure its relevance, clarity, and comprehensiveness. Cronbach's alpha coefficient was used to check the reliability of the questionnaire, which was 90.4% in the “educational information” category, 91.8% in the “disease and complications management” category, 90.2% in the “patient profile” category, and 74.1% in the “technical capabilities” category. Although the alpha for Technical Capabilities was lower than the other categories, it still indicates acceptable internal consistency. This lower value may be due to the broader range of technical features assessed, which are inherently more diverse. Overall, the reliability of all categories was satisfactory for the purposes of this study.

Participants were recruited using purposive sampling to ensure that individuals with relevant experience and knowledge were included. The patient sample consisted of 20 individuals with spinal cord injury (complete/incomplete) who were capable of using mobile phones. These patients were selected from Nasim Clinic and Shohada Tajrish Hospital, which serve a diverse patient population regarding cultural and geographical backgrounds. The specialist sample included 20 healthcare professionals with at least 5 years of SCI care experience, including neurosurgeons(*n* = 11), physiotherapists(*n* = 3), and nurses(*n* = 6) in neurosurgery departments. Purposive sampling was employed to intentionally select participants who could provide in‐depth insights into the data and functional requirements for the mobile application. This method was chosen to enhance the study's reliability by capturing a broad range of perspectives relevant to SCI self‐management. The validated questionnaire was distributed in person to participants using a purposive sampling method. The participants were asked to rate each item in the questionnaire (with five being very important and one being unimportant). The item ratings were as follows: 5 = very important, 4 = important, 3 = no idea, 2 = slightly important, and 1 = unimportant.

### 2.4 Statistical Analysis

Data analysis was performed using SPSS version 26. Descriptive statistics were used to summarize the demographic characteristics of the participants, including frequencies and percentages for categorical variables (e.g., gender, marital status, profession) and means for continuous variables (e.g., age, years of experience). For the questionnaire data, descriptive statistics were used to assess the importance ratings of each item. Specifically, means were calculated for each item within the “educational information”, “disease and complication management”, “patient profile”, and “technical capabilities” categories. Frequency distributions were also examined to understand the distribution of responses for each item on the 5‐point Likert scale. These analyses allowed for the identification of key requirements based on the average importance scores assigned by both patients and specialists.

### 2.5 Ethical Considerations

This article is a part of the master's thesis project titled “Development of a Mobile Application for Self‐management of Spinal Cord Injury Patients”, approved in 2024, which was reviewed and approved by the review board and ethics committee of Shahid Beheshti University of Medical Sciences. (IR.SBMU.RETECH.REC.1402.858). Informed consent was obtained from all participants involved in the study.

## 3 Results

Demographic information on the patients with spinal cord injury (SCI) and the clinical specialists who participated in the study is presented in Tables 2 and 3. Among the clinical specialists, 13 (65%) were male, and 10 (50%) had 5–10 years of work experience. Additionally, 11 (55%) of the specialists were over 40. Among the specialists, 11 (55%) were neurosurgeons, 3 (15%) were physiotherapists, and 6 (30%) were nurses working in neurosurgery departments. Although neurosurgeons were overrepresented in our sample, it is important to recognize their crucial role in the acute management and initial stabilization of SCI. Their input was valuable in identifying key requirements related to early complications and postoperative care. However, the absence of physiatrists and rehabilitation specialists necessitates caution when generalizing these findings to the long‐term self‐management needs of SCI patients. In the SCI patient group, 16 (80%) patients were male and 4 (20%) patients were female, with 15 (75%) being single. Furthermore, 9 (45%) of the patients had suffered spinal cord injury within the past 1–5 years.

**Table 2:** Frequency and demographic data of the clinical specialists included in the study.

Variables	Number (*n*)	Frequency (%)
Gender		
Man	13	65
Woman	7	35
Age (years)		
< 30	3	15
30–40	6	30
40–50	8	40
50–60	3	15
Work experience (years)		
< 5	3	15
5–10	10	50
10–15	4	20
15–20	2	10
> 20	1	5
Occupation		
Neurosurgery subspecialty	3	15
Neurosurgery specialty	8	40
Nurse	6	30
Physiotherapist	3	15
Total	20	100

**Table 3:** Frequency and demographic data of the patients in the study.

Variables	Number (*n*)	Frequency (%)
Gender		
Man	16	80
Woman	4	20
Marital status		
Single	15	75
Married	5	25
Age (years)		
20–30	7	35
30–40	8	40
40–50	4	20
50–60	1	5
Level of education		
High school	4	20
Diploma	5	25
Associate degree	1	5
BSc	5	25
MSc	5	25
PhD	0	0
Duration of spinal cord injury		
Less than a year	6	30
1–5 years	9	45
5–10 years	3	15
More than 10 years	2	10
Total	20	100

The key requirements for developing a mobile application for the self‐management of SCI patients were identified based on the findings. The proposed data requirements were categorized into three thematic categories: “educational information” (17 items), “patient profile” (10 items), and “disease and complication management” (7 items). The proposed functional requirements fall under the “technical capabilities” category (9 items). The expert panel validated these as key requirements for developing self‐management applications. Table 4 presents the data requirements in the “patient profile” category. From the perspectives of specialists and patients, the “Type of SCI” (with an average score of 4.80 out of 5) received the highest rating, and “Height” (with an average score of 4.22 out of 5) received the lowest rating.

**Table 4:** Distribution of participants' responses in the category of patient profile.

No.	Data requirements	Clinical specialists (mean)	Patients (mean)	Total (mean)	%
1	First and last name	4.50	4.20	4.35	87
2	Gender	4.70	4.50	4.60	92
3	Age	4.65	4.25	4.45	89
4	Marital status	4.20	4.40	4.30	86
5	Duration of SCI	4.85	4.70	4.77	95.5
6	Height	4.25	4.20	4.22	84.5
7	Weigh	4.35	4.35	4.35	87
8	Occupation	4.25	4.50	4.37	87.5
9	Level of education	4.35	4.45	4.40	88
10	Type of SCI	4.90	4.70	4.80	96
	Average	4.50	4.42	4.46	89.2

Based on the data presented in Table 5 Distribution of participants' responses in the category of educational information. In the “disease and complication management” category, “rehabilitation management” (with an average score of 4.75 out of 5) had the highest score, and “complementary therapies” (with an average score of 4.40 out of 5) had the lowest score. Table 5 shows the data requirements in the “disease and complication management” category.

**Table 5:** Distribution of participants' responses in the category of disease and complications management.

No.	Data requirements	Clinical specialists (mean)	Patients (mean)	Total (mean)	%
1	About the SCI	4.60	4.40	4.50	90
2	Complementary therapies	4.45	4.35	4.40	88
3	Medication	4.50	4.40	4.45	89
4	Rehabilitation management	4.70	4.80	4.75	95
5	Complications and clinical manifestations	4.60	4.50	4.55	91
6	Common issues for patients	4.55	4.65	4.65	93
7	Routine tests to diagnose complications of SCI	4.55	4.50	4.52	90.5
	Average	4.56	4.51	4.53	90.6

Table 6 presents the data requirements in the “educational information” category. Table 6 shows in this category, “appropriate movement positions to prevent pressure ulcers” (with an average score of 4.85 out of 5) had the highest score, whereas “body temperature control” (with an average score of 4.27 out of 5) had the lowest score.

**Table 6:** Distribution of participants' responses in the category of educational information.

No.	Data requirements	Clinical specialists (mean)	Patients (mean)	Total (mean)	%
1	Skincare and pressure sores	4.80	4.80	4.80	96
2	Mental health management	4.80	4.70	4.75	95
3	Weight control	4.45	4.55	4.50	90
4	Chronic pain management	4.65	4.30	4.47	89.5
5	Management of respiratory complications	4.70	4.50	4.60	92
6	Managing activities of daily living	4.50	4.60	4.55	91
7	Nutrition management and diet plan	4.70	4.80	4.75	95
8	Training to use a wheelchair	4.70	4.80	4.75	95
9	Urine and feces excretion protocol	4.60	4.65	4.62	92.4
10	Training self‐catheterization in men and women	4.60	4.60	4.60	92
11	Bowel and bladder management	4.70	4.60	4.65	93
12	Body temperature control	4.45	4.10	4.27	85.4
13	Exposure management of autonomic dysreflexia	4.70	4.60	4.65	93
14	Training to use a catheter condom	4.65	4.55	4.60	92
15	Sexual health after SCI	4.70	4.90	4.80	96
16	Appropriate movement positions to prevent pressure ulcers	4.80	4.90	4.85	97
17	Frequently asked questions (FAQ)	4.40	4.50	4.45	89
	Average	4.64	4.61	4.63	92.6

Based on the data presented in Table 7 In the “technical capabilities” category, “medication reminder” (with an average score of 4.80 out of 5) had the highest score, and “list of rehabilitation centers” (with an average score of 4.40 out of 5) had the lowest score. Table 7 presents the functional requirements in the “technical capabilities” category.

**Table 7:** Distribution of participants' responses in the category of technical capabilities.

No.	Technical features	Clinical specialists (mean)	Patients (mean)	Total (mean)	%
1	Collecting data	4.60	4.80	4.70	92
2	Web‐based	4.50	4.50	4.50	90
3	Follow up	4.90	4.60	4.75	95
4	Patient's notebook	4.35	4.55	4.45	89
5	Appointment reminder	4.60	4.40	4.50	90
6	Medication reminder	4.75	4.85	4.80	96
7	A reminder to do rehabilitation exercises	4.60	4.40	4.50	90
8	Providing a list of specialist doctors	4.25	4.65	4.45	89
9	Providing a list of rehabilitation centers	4.20	4.60	4.40	88
	Average	4.53	4.59	4.56	91.2

**Figure 3:**
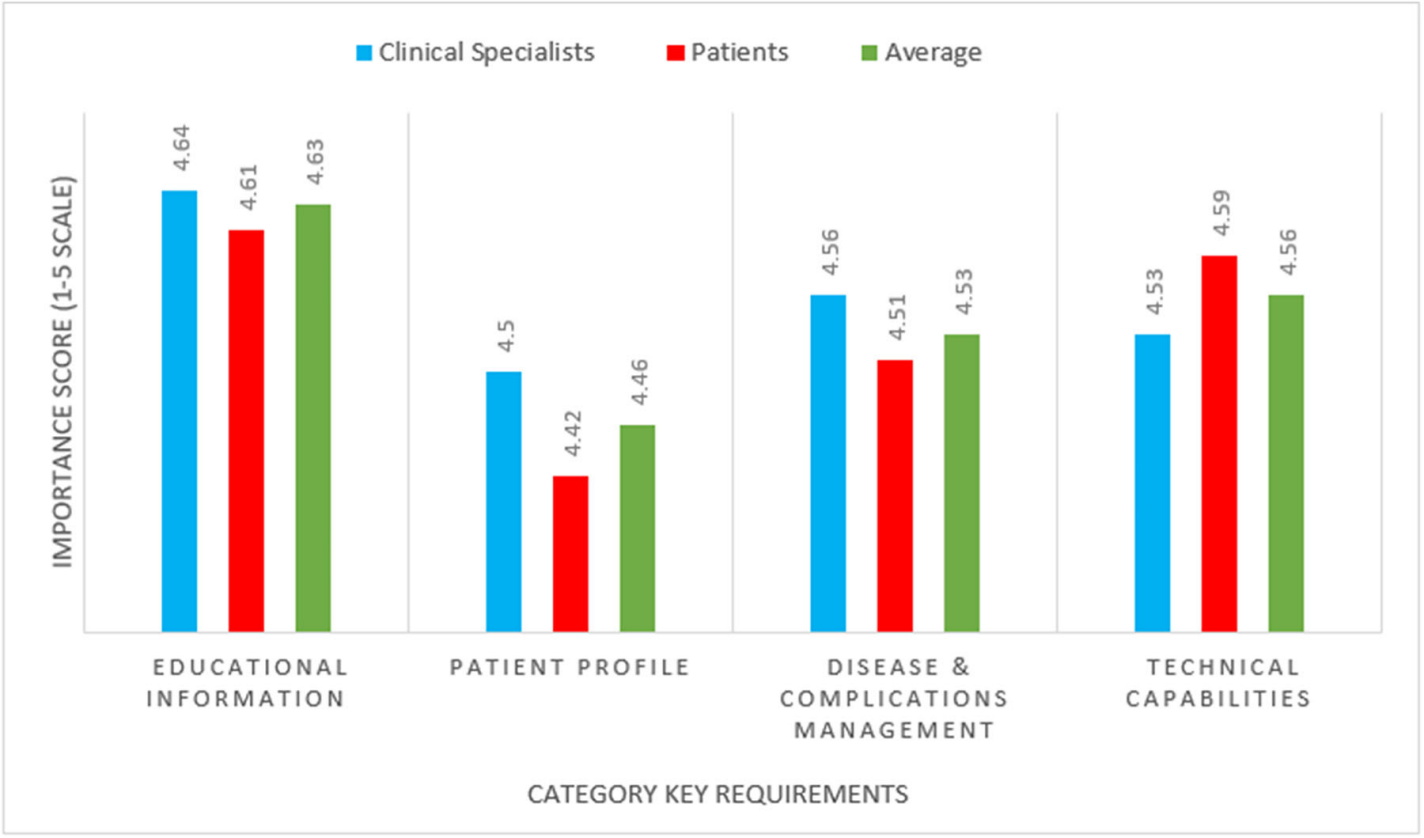
Average scores of key requirements in each category based on responses from clinical specialists and patients.

Four key requirement categories for developing mobile application self‐management for SCI patients were analyzed and compared (Figure 3). The “educational information” category had the highest average score (4.63 out of 5), whereas the “patient profile” category had the lowest average score (4.46 out of 5) among the key requirement categories. The results from Figure 3 show that most patients and specialists viewed the “patient profile” category with less importance and agreement than the other categories. On the other hand, the “educational information” category was considered of more important and received more favorable feedback from both patients and specialists.

Although the questionnaire included an item on injury completeness (complete vs. incomplete), detailed information on the neurological level of injury (e.g., cervical vs. thoracic) was not systematically recorded in the medical records, and most participants were unaware of their specific injury level. Therefore, stratification by tetraplegia/paraplegia was not feasible in this study. To partially address the issue of injury severity, we conducted a secondary analysis comparing self‐management requirements between participants with complete and Incomplete injuries. There was no significant difference in the “patient profile” category and “technical capabilities” between complete and incomplete spinal cord injuries. The categories of “educational information”, and “disease and complication management” are given more importance by spinal cord injury patients with complete injuries compared to those with incomplete spinal cord injury (Table 8).

**Table 8:** Comparison of mean scores for S–M requirement categories between patients with complete and incomplete SCI (Likert scale 1–5).

No.	Key requirement	Complete SCI (mean)	Incomplete SCI (mean)
1	Patient profile	4.40	4.40
2	Educational information	4.80	4.40
3	Disease and complications management	4.60	4.40
4	Technical capabilities	4.60	4.58

## 4 Discussion

The use of self‐management mobile applications that respond to the information and educational needs of these patients is necessary to empower patients with spinal cord injury and the need to use accompanying health capacities in health care. For this purpose, it is essential to identify key requirements, including data and functional requirements, as the first phase in developing a self‐management mobile application for patients with spinal cord injury.

To the best of our knowledge, this is the first study to identify and analyze the key requirements for mobile applications for SCI patients. Additionally, it is one of the few studies that consider both the perspectives of specialists and patients in developing a mobile application self‐management application for SCI.

Mobile health apps for the self‐management of patients with SCI can improve patients' ability to take care of themselves and, as a result, increase their quality of life, reduce the rehospitalization rate, and prevent adverse outcomes [30, 31, 32].

Identifying data and functional requirements in designing self‐management mobile applications based on the needs of spinal cord injury patients and the views of experts in this field can lead to providing an educational program and help to better identify the educational and informational needs of these patients.

The present study's findings indicated that the “educational information” category, with an average score of 4.63 out of 5, was the most important among the data requirements for developing a self‐management mobile application for patients with SCI. These results emphasize that acquiring educational information is very important from the perspective of both patients and specialists. In developing a self‐management mobile application for SCI patients, providing accurate information and proper education can reduce the information gap about their condition and help meet their self‐management needs.

Pressure ulcers are one of the most distressing complications faced by SCI patients, which can develop after hospital admission, prolong treatment, lead to social withdrawal, and exacerbate mobility restrictions [33]. Therefore, among the data requirements in the “educational information” category, “appropriate movement positions to prevent pressure ulcers” (with an average score of 4.85 out of 5) and “skincare and pressure sores” (average score of 4.80 out of 5) received the highest scores from patients and specialists, confirming the inclusion of these two data requirements in the “educational information” category.

Mental health problems, particularly depression, are also common among SCI patients, with an estimated 25%–30% of them experiencing depression. For this reason, “mental health management” was identified as a high‐priority data requirement within the “educational information” category, receiving a score of 4.75 out of 5 from both patients and specialists [34].

The study also revealed the importance of data requirements for managing SCI and its numerous associated complications. Data that provides information on SCI, complementary therapies, side effects, common problems, and routine diagnostic tests are essential. As such, “rehabilitation management” scored the highest (4.75 out of 5) in the “disease and complications management” category.

The data requirements for the “patient profile” category align with the demographic requirements identified in similar studies that have developed mobile applications [35, 36, 37]. Both specialists and patients emphasized the importance of data exchange, communication with healthcare providers, and patient follow‐up using mobile applications. As a result, they regarded the “patient profile” data requirements as highly significant for developing a self‐management mobile application for SCI patients. Initially, the “patient profile” category included national ID, insurance type, and place of birth as data items. However, after review by the expert panel, these three items did not score highly enough and were excluded from the final list.

All functional requirements identified in the “technical capabilities” category received high scores for inclusion in the self‐management mobile application. “Medication reminder was rated the highest, with an average score of 4.8 out of 5 from specialists and SCI patients. This highlights the significance of this technical feature, which is likely to improve medication adherence among SCI patients.

In the study conducted by Sheikhtaheri et al. [37], which aimed to identify the requirements for self‐management mobile applications for colorectal cancer patients, features such as “medication reminder” and “doctor appointment reminder,” similar to the present study were identified as essential requirements in designing a self‐management application.

In 2022, another study conducted by Tadayon et al. [38] aimed to determine the functional requirements of a self‐management mobile application for stroke survivors. This study identified skin care, bladder, and bowel problems, daily activities, medication reminders, doctor appointment reminders, and rehabilitation as key requirements in designing a self‐management mobile application for stroke patients, like the present research.

In 2022, a study by Yazdanyan et al. [19] was conducted with the aim of identifying user requirements for developing a mobile self‐management application to support gastric cancer patients. In this study, through two rounds of the Delphi technique, data requirements were categorized into demographic data, clinical data, disease management, educational information, and functional requirements, which were divided into notifications, alerts, reminders, and display capabilities.

In another study [36] conducted in 2017 to identify educational and informational components for developing a self‐management mobile application for psoriasis patients, educational information, lifestyle management, clinical and demographic data, and the application capabilities of the self‐management application were recognized as necessary components for developing a self‐management mobile application for psoriasis patients.

One of the strengths of the present study is the Participation of spinal cord injury patients, who are the primary users of the mobile health application. Another strength is the participation and consultation of a multidisciplinary team, including neurosurgeons, nurses, physiotherapists, health information technology experts, health information management specialists, and medical informatics specialists.

It is recommended that designers and developers of mobile health apps consider the key requirements identified in our research when developing future applications. Future studies could also evaluate the effectiveness of the self‐management mobile application developed in our research or similar health platforms.

This study offers valuable conceptual ideas for developing mobile health applications for other diseases. It can also assist the Ministry of Health and Medical Education of Iran, as well as other healthcare centers involved in caring for individuals with spinal cord injury, in better identifying the needs of these patients, which would provide more accessible tools to improve the quality of life for patients with spinal cord injury and enhance the quality of healthcare services offered.

### 4.1 Limitations

This study has several limitations. First, only English‐language self‐management sources were reviewed, possibly omitting relevant evidence in other languages. Second, the small sample size (20 patients from two centers in Tehran) limits the generalizability of the findings. Third, the lack of systematic documentation of neurological injury level prevented subgroup analysis by tetraplegia and paraplegia, reducing the clinical specificity of the results. We recommend that future studies collect detailed injury data to enable more tailored interventions. Finally, the specialist panel was dominated by neurosurgeons, while perspectives from rehabilitation and allied health professionals were limited. Future research should involve a more diverse range of specialists to better address the full spectrum of self‐management needs in SCI care.

## 5 Conclusion

This study identified the data and functional requirements for developing a self‐management mobile application for patients with spinal cord injury. The findings emphasize the importance of designing and developing a well‐tailored application based on pre‐identified needs. Such an application could improve the quality of life and enhance the empowerment of these patients in managing their health conditions. The key requirements identified in this study can be used to determine the educational content and design of a self‐management mobile application for spinal cord injury.

## Author Contributions

**Amir Hossein Daeechini:** conceptualization, methodology, writing – review and editing, writing – original draft, and data curation. **Azamossadat Hosseini:** supervision, methodology, conceptualization, writing – review and editing. **Reza Rabiei:** investigation and validation. **Saeed Oraee‐Yazdani:** data curation and investigation. **Somayeh Paydar:** data curation, validation, and investigation. **Fatemeh Rahimi:** writing – review and editing, validation, and investigation.

## Ethics Statement

This article is a part of the master's thesis project titled “Development of a Mobile Application for Self‐management of Spinal Cord Injury Patients”, approved in 2024, which was reviewed and approved by the review board and ethics committee of Shahid Beheshti University of Medical Sciences. (IR.SBMU.RETECH.REC.1402.858).

## Consent

Informed consent was obtained from all participants involved in the study.

## Conflicts of Interest

The authors declare no conflict of interest.

## Transparency Statement

The lead author Azamossadat Hosseini affirms that this manuscript is an honest, accurate, and transparent account of the study being reported; that no important aspects of the study have been omitted; and that any discrepancies from the study as planned (and, if relevant, registered) have been explained.

## Supporting information

Supplementary file1 amp 2 2.

## Data Availability

The authors confirm that the data supporting the findings of this study are available within the article (and/or) its supporting information.
