# Supplementary material for: Development of a Mobile Application for the Self‐Management of Spinal Cord Injury Patients: Identification and Analysis of Key Requirements
Source: Health Sci Rep. 2025 Nov 9;8(11):e71340. doi: 10.1002/hsr2.71340 (PMC12598095; doi:10.1002/hsr2.71340)
Supplement: Supplementary file 1 — Supplementary file1 amp 2 2. [file HSR2-8-e71340-s001.docx]

**Appendix A (Patient)**

**Supplementary 1. Questionnaire Key Requirements for Mobile Application for the Self-Management of Spinal Cord Injury Patients**

Dear respondents,

The present questionnaire is related to the master's thesis entitled " Development of a Mobile Application for Self-management of Spinal Cord Injury Patients". Your careful answer will help achieve the thesis's objectives and determine the content for creating an application. The time to answer the questionnaire is about 25 to 30 minutes. Remember that your answers will remain confidential to the researcher. Please answer the following questions according to the level of importance (very important=5, important=4, no Idea=3, slightly Important=2, unimportant=1).

Thank you in advance for your cooperation.

Amir Hossein Daeechini

Email: [amir.h.daeechini1377@sbmu.ac.ir](mailto:amir.h.daeechini1377@sbmu.ac.ir)

**Part 1: Demographic data (Patient)**

Please fill in this section:

1) Gender:

2) Age (y):

3) Levels of education:

4) Marital statuses:

5) Duration of spinal cord injury:

6) Complete/Incomplete spinal cord injury:

**Part 2: Data and functional requirements**

| **Data and functional requirements** | | **Likert scale** | | | | |
| --- | --- | --- | --- | --- | --- | --- |
|  |  | **very Important**  **(5)** | **Important**  **(4)** | **no Idea**  **(3)** | **slightly Important**  **(2)** | **unimportant**  **(1)** |
| **Patient profile** | **First & last name** |  |  |  |  |  |
|  | **Gender** |  |  |  |  |  |
|  | **Age** |  |  |  |  |  |
|  | **Marital status** |  |  |  |  |  |
|  | **Duration of SCI** |  |  |  |  |  |
|  | **Height** |  |  |  |  |  |
|  | **Weigh** |  |  |  |  |  |
|  | **Occupation** |  |  |  |  |  |
|  | **Level of Education** |  |  |  |  |  |
|  | **Type of SCI** |  |  |  |  |  |
| **Disease and complications management** | **About the SCI** |  |  |  |  |  |
|  | **Complementary therapies** |  |  |  |  |  |
|  | **Medication** |  |  |  |  |  |
|  | **Rehabilitation Management** |  |  |  |  |  |
|  | **Complications & clinical manifestations** |  |  |  |  |  |
|  | **Management of Respiratory Complications** |  |  |  |  |  |
|  | **Routine tests to diagnose complications of SCI** |  |  |  |  |  |
| **Educational**  **information** | **Skincare & Pressure Sores** |  |  |  |  |  |
|  | **Mental Health Management** |  |  |  |  |  |
|  | **Weight Control** |  |  |  |  |  |
|  | **Chronic Pain Management** |  |  |  |  |  |
|  | **Common issues for patients** |  |  |  |  |  |
|  | **Managing Activities of Daily Living** |  |  |  |  |  |
|  | **Nutrition Management & Diet Plan** |  |  |  |  |  |
|  | **Training to use a Wheelchair** |  |  |  |  |  |
|  | **Urine & feces excretion protocol** |  |  |  |  |  |
|  | **Training self-catheterization in men and women** |  |  |  |  |  |
|  | **Bowel & Bladder Management** |  |  |  |  |  |
|  | **Body temperature control** |  |  |  |  |  |
|  | **Exposure Management of Autonomic Dysreflexia** |  |  |  |  |  |
|  | **Training to Use a Catheter condom** |  |  |  |  |  |
|  | **Sexual health after SCI** |  |  |  |  |  |
|  | **Appropriate Movement positions to prevent Pressure Ulcers** |  |  |  |  |  |
|  | **Frequently Asked Questions (FAQ)** |  |  |  |  |  |
| **Technical capabilities** | **Collecting data** |  |  |  |  |  |
|  | **Web-based** |  |  |  |  |  |
|  | **Follow up** |  |  |  |  |  |
|  | **Patient's notebook** |  |  |  |  |  |
|  | **Appointment reminder** |  |  |  |  |  |
|  | **Medication reminder** |  |  |  |  |  |
|  | **A reminder to do rehabilitation exercises** |  |  |  |  |  |
|  | **Providing a list of specialist doctors** |  |  |  |  |  |
|  | **Providing a list of rehabilitation centers** |  |  |  |  |  |

**Part 3: Please enter your suggestions below**

………………………………………………………………………………………………………………………………………………………………………………………………………………………………………………………………………………………………………………………………………………

**Appendix B (Clinical Specialists)**

**Supplementary 2. Questionnaire Key Requirements for Mobile Application for the Self-Management of Spinal Cord Injury Patients**

Dear respondents,

The present questionnaire is related to the master's thesis entitled " Development of a Mobile Application for Self-management of Spinal Cord Injury Patients". Your careful answer will help achieve the thesis's objectives and determine the content for creating an application. The time to answer the questionnaire is about 25 to 30 minutes. Remember that your answers will remain confidential to the researcher. Please answer the following questions according to the level of importance (very important=5, important=4, no Idea=3, slightly Important=2, unimportant=1).

Thank you in advance for your cooperation.

Amir Hossein Daeechini

Email: [amir.h.daeechini1377@sbmu.ac.ir](mailto:amir.h.daeechini1377@sbmu.ac.ir)

**Part 1: Demographic data** (**Clinical Specialists)**

**Please fill in this section:**

1) Gender:

2) Age (y):

3) Occupation:

4) Work Experience (y):

**Part 2: Data and functional requirements**

| **Data and functional requirements** | | **Likert scale** | | | | |
| --- | --- | --- | --- | --- | --- | --- |
|  |  | **very Important**  **(5)** | **Important**  **(4)** | **no** **Idea**  **(3)** | **slightly Important**  **(2)** | **unimportant**  **(1)** |
| **Patient profile** | **First & last name** |  |  |  |  |  |
|  | **Gender** |  |  |  |  |  |
|  | **Age** |  |  |  |  |  |
|  | **Marital status** |  |  |  |  |  |
|  | **Duration of SCI** |  |  |  |  |  |
|  | **Height** |  |  |  |  |  |
|  | **Weigh** |  |  |  |  |  |
|  | **Occupation** |  |  |  |  |  |
|  | **Level of Education** |  |  |  |  |  |
|  | **Type of SCI** |  |  |  |  |  |
| **Disease and complications management** | **About the SCI** |  |  |  |  |  |
|  | **Complementary therapies** |  |  |  |  |  |
|  | **Medication** |  |  |  |  |  |
|  | **Rehabilitation Management** |  |  |  |  |  |
|  | **Complications & clinical manifestations** |  |  |  |  |  |
|  | **Management of Respiratory Complications** |  |  |  |  |  |
|  | **Routine tests to diagnose complications of SCI** |  |  |  |  |  |
| **Educational**  **information** | **Skincare & Pressure Sores** |  |  |  |  |  |
|  | **Mental Health Management** |  |  |  |  |  |
|  | **Weight Control** |  |  |  |  |  |
|  | **Chronic Pain Management** |  |  |  |  |  |
|  | **Common issues for patients** |  |  |  |  |  |
|  | **Managing Activities of Daily Living** |  |  |  |  |  |
|  | **Nutrition Management & Diet Plan** |  |  |  |  |  |
|  | **Training to use a Wheelchair** |  |  |  |  |  |
|  | **Urine & feces excretion protocol** |  |  |  |  |  |
|  | **Training self-catheterization in men and women** |  |  |  |  |  |
|  | **Bowel & Bladder Management** |  |  |  |  |  |
|  | **Body temperature control** |  |  |  |  |  |
|  | **Exposure Management of Autonomic Dysreflexia** |  |  |  |  |  |
|  | **Training to Use a Catheter condom** |  |  |  |  |  |
|  | **Sexual health after SCI** |  |  |  |  |  |
|  | **Appropriate Movement positions to prevent Pressure Ulcers** |  |  |  |  |  |
|  | **Frequently Asked Questions (FAQ)** |  |  |  |  |  |
| **Technical capabilities** | **Collecting data** |  |  |  |  |  |
|  | **Web-based** |  |  |  |  |  |
|  | **Follow up** |  |  |  |  |  |
|  | **Patient's notebook** |  |  |  |  |  |
|  | **Appointment reminder** |  |  |  |  |  |
|  | **Medication reminder** |  |  |  |  |  |
|  | **A reminder to do rehabilitation exercises** |  |  |  |  |  |
|  | **Providing a list of specialist doctors** |  |  |  |  |  |
|  | **Providing a list of rehabilitation centers** |  |  |  |  |  |

**Part 3: Please enter your suggestions below**

………………………………………………………………………………………………………………………………………………………………………………………………………………………………………………………………………………………………………………………………………………
